# Dual-resolution megahertz optical coherence tomography prototype rectoscope for enhanced visualization of colorectal microstructures

**DOI:** 10.1117/1.JBO.31.4.046002

**Published:** 2026-03-27

**Authors:** Sazgar Burhan, Berenice Schulte, Madita Göb, Awanish Pratap Singh, Bayan Mustafa, Simon Lotz, Wolfgang Draxinger, Philipp Lamminger, Yasmeine Saker, Tim Eixmann, Martin Ahrens, Marvin Heimke, Tillmann Heinze, Thilo Wedel, Maik Rahlves, Mark Ellrichmann, Robert Huber

**Affiliations:** a Universität zu Lübeck, Institute of Biomedical Optics, Lübeck, Germany; b Medizinisches Laserzentrum Lübeck GmbH, Lübeck, Germany; c University Hospital Schleswig-Holstein, Interdisciplinary Endoscopy, Medical Department I, Kiel, Germany; d Kiel University, Institute of Anatomy, Center of Clinical Anatomy, Kiel, Germany; e University of Oldenburg, University Clinic for Internal Medicine – Gastroenterology, Hepatology, Metabolic Medicine, Renal and Hypertensive Diseases, Oldenburg, Germany

**Keywords:** MHz optical coherence tomography, rectoscope, dual mode design, extended-range imaging mode, high-detail imaging mode, ethanol-glycerol-lysoformin fixation

## Abstract

**Significance:**

Endoscopic optical coherence tomography (OCT) is a valuable tool for rectal imaging, enabling noninvasive visualization of transmural structures with near-histological resolution, critical for accurate tumor staging and diagnostic/therapeutic monitoring. However, anatomical variability of the rectum and the resulting changes in tissue-to-probe distance challenge optimal circumferential imaging.

**Aim:**

We aim to develop a dual-mode endoscopic OCT system, enabling real-time switching between high-detail and extended-range imaging for improved assessment of rectal wall morphology under varying anatomical conditions.

**Approach:**

A single fiber-optic ferrule containing two fibers with differing mode fields was integrated into a MHz-OCT rectoscope, enabling an extended-range mode for full circumferential imaging and a high-detail mode for close examination of fine structures with seamless live switching mechanism. The system was tested *in situ* on a postmortem human rectum preserved with ethanol-glycerol-lysoformin fixation.

**Results:**

Postmortem rectum imaging demonstrated the feasibility of real-time switching between the two modes. Extended-range imaging provided full circumferential coverage, whereas high-detail imaging revealed distinct rectal wall layers and fine transmural structures, validated by histological correlation.

**Conclusions:**

The dual-mode MHz-OCT system offers a flexible and practical solution for adaptive rectal imaging, providing high-resolution detail and full circumferential coverage with the potential to enhance diagnostics and treatment monitoring.

## 1 Introduction

Colorectal cancer is the third most common and the second deadliest cancer worldwide, causing over 900,000 deaths annually.^1^ Early and accurate tumor staging is a key factor in reducing mortality, which allows for timely and effective treatment interventions. Physicians rely on various imaging techniques to assess tumor infiltration depth (T-staging), commonly utilizing colonoscopy, histology, magnetic resonance imaging, and endoscopic ultrasound (EUS). However, accurately assessing the tumor’s infiltration depth remains challenging due to the limited resolution of EUS, particularly given the structural complexity of the rectal wall, including four layers with a median thickness of ∼2.6  mm.^2^ The mucosa forms the innermost layer, delimited by the lamina muscularis mucosae, followed by the submucosa and the muscularis propria, comprising the circular and longitudinal muscle layers. Depending on the location, the outermost layer of the rectum corresponds to the serosa or the adventitia. The imaging system for grading rectal tumors must accurately differentiate between these layers. As the axial resolution ranges from 200 to 300  μm, EUS often faces limitations in exact discrimination, as the carcinoma *in situ* (Tis) is restricted to the mucosa, whereas T1 infiltrates the submucosa and T2 reaches into the muscularis propria.^3^ Borderline findings occur when EUS cannot exactly discriminate whether the next wall layer is reached or exceeded, leading to over- or under-staging.

Optical coherence tomography (OCT) is a noninvasive imaging technique based on the principles of low-coherence interferometry. It measures the time-of-flight delay of the backscattered light relative to a reference reflector to acquire depth resolved images. Current systems typically achieve an imaging depth of 1 to 3 mm in scattering tissue, with an axial resolution of ∼10  μm and a lateral resolution of ∼20  μm, depending on the specific setup.^4^ Given that the thickness of the rectal wall tissue falls within the imaging range of OCT, it is well-suited for rectal applications.

In gastroenterology, OCT endoscopy is a promising technique to assess the rectum.^5^^–^^12^ Endoscopic OCT systems based on novel probe designs already enabled the visualization of transmural structures in different anatomical regions, which has led to the development of a number of endoscope types matched to the medical requirements.^6^^,^^13^^–^^20^ Among these attempts, the NvisionVLE Imaging System was licensed for clinical use in the upper gastrointestinal tract and was applied to image the esophageal wall, performing circumferential scanning of 6 cm longitudinal segments within 90 s. The system was also evaluated in a clinical trial for colon imaging, where it was tested for the detection and characterization of colorectal lesions.^20^ Although this system showed promise in distinguishing high-grade lesions from polyps, it is no longer commercially available, and the slow imaging speed restricted its applicability for scanning larger areas. For instance, scanning the full length of the rectum (up to 16 cm) would take ∼4  min. Extending imaging to the entire colon (∼150  cm), a potential future application, would require nearly 37.5 min, making it impractical for routine clinical use. Such long acquisition times pose challenges for real-time imaging, reduce patient comfort, and limit the feasibility of comprehensive colon assessment.

One approach to address this limitation is using a light source with MHz sweep rates.^21^ In our previous study, we demonstrated that a swept-source OCT system incorporating a 3.2 MHz Fourier domain mode locking (FDML) laser^22^ achieved a scanning speed of ∼5 to 10  mm/s, with a B-scan frame rate of 667 Hz and a spot size of 19  μm.^9^ With this setup, the imaging time could be markedly reduced compared with the NvisionVLE Imaging System, and the entire rectum could be theoretically captured in an average time of 20.25 s.

Another challenge to address is the varying diameter of the rectum. As the distance between the rectoscope, positioned in the center of the lumen, and the tissue wall can vary by several millimeters during a circular scan, a 19  μm spot size is inadequate. Its short Rayleigh length limits the focal range to a few hundred micrometers, causing a rapid signal drop-off outside this region. By contrast, a longer Rayleigh length comes at the expense of transverse resolution, which can hinder the precise detection of fine details in the wall layers. For instance, Adler et al.^11^ demonstrated that a transverse spot size of 20  μm is necessary to visualize crypt structures effectively. This trade-off between a large depth of focus and high transverse resolution remains a major challenge.

Switching between multiple rectoscopes with varying imaging capabilities would address this issue. Nevertheless, due to longer examination times and higher costs, it is inconvenient for clinicians and patients, reducing its practicality for routine clinical use. This highlights the need for a system that seamlessly switches between an extended Rayleigh range and a high transverse resolution mode, enabling an initial comprehensive scan followed by detailed imaging of specific areas of interest.

Building on the previous rectoscope platform,^9^^,^^14^ the present work introduces a dual-resolution MHz-OCT rectoscope based on a novel dual-fiber approach and micro-electromechanical system (MEMS)-based switch. Two fiber channels with different effective mode field diameters (MFD) are integrated within a single fiber optic ferrule. The first channel, the high-detail mode (HD), incorporates a standard single-mode fiber (SMF), yielding a small mode field diameter (MFD) and thereby high transverse resolution for detailed structural assessment. The second channel, the extended-range mode (ER), integrates a specifically engineered fiber assembly using coreless and gradient-index (GRIN) fibers to enlarge the MFD, producing an increased spot size and extended Rayleigh length for comprehensive circumferential imaging of the undulating rectal wall. A MEMS fiber switch integrated into the endoscope allows seamless, real-time selection between the two spot sizes depending on the selected channel. Although the first demonstrator of this dual-resolution concept was presented in a preliminary conference abstract,^23^ the present manuscript provides a comprehensive characterization of the system, demonstrates *in situ* OCT imaging in a body donor’s rectum, and validates these findings through histological correlation.

## 2 Methods

### 2.1 Design Considerations of the extended-range mode

**Fig. 1:**
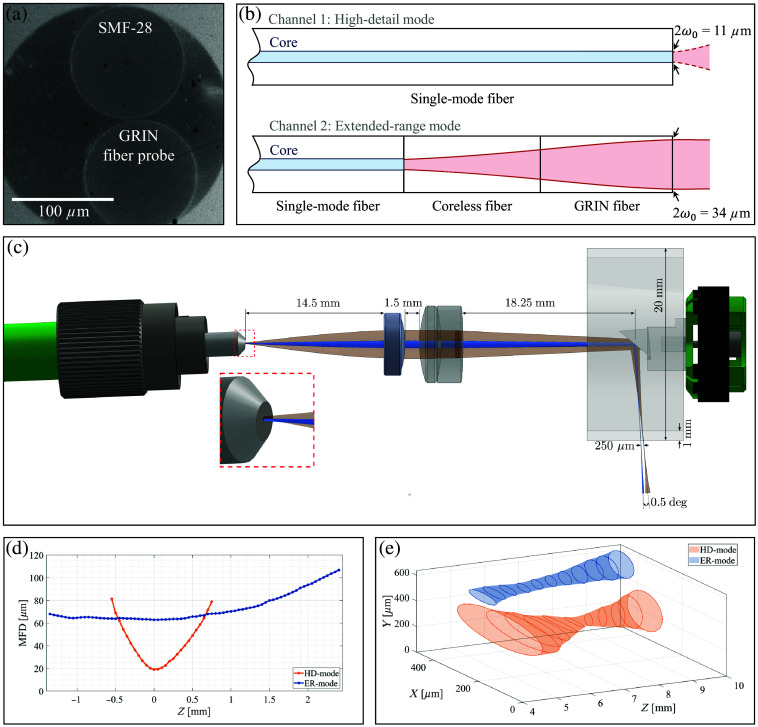
Setup and beam characteristics of the fiber-optic ferrule. (a) Front view of the ferrule showing the two fiber channels: the upper circle corresponds to the single-mode fiber (SMF-28), and the lower circle to the GRIN fiber probe. (b) Schematic illustration of light propagation in the two fiber channels and resulting mode field diameters (MFD, $2\omega_{0}$) at the ferrule tip. (c) 3D CAD rendering of beam propagation for both imaging modes in the distal rectoscope tip [HD, high-detail mode (orange); ER, extended-range mode (blue)]. The actual beam profiles in the focal region are shown in panels (d) and (e). (d) 2D beam profile with measured MFDs at multiple axial positions (Z) around the waist after the scan lens. (e) 3D geometry of the two beam waists through the fully assembled distal tip.

The ER mode is designed to achieve comprehensive circumferential imaging of the rectal wall by increasing the effective MFD and thereby extending the Rayleigh length, as illustrated in Fig. 1. This is achieved by fusion-splicing a coreless and a short section of GRIN fiber to the end of the OCT’s SMF. This well-established configuration is employed to modify the output MFD of the fiber^18^^,^^24^^–^^27^ and, in this implementation, serves to reduce the numerical aperture (NA), acceptance angle, and light divergence, thereby maintaining a focused beam over extended distances. Adjusting the lengths of the coreless spacer and GRIN fiber enables precise control of the NA and imaging range, forming the basis of the “GRIN fiber probe” configuration described below. The coreless fiber (FG125LA, Thorlabs Inc., USA) is used as a spacer between the SMF (SMF–28e+, Corning Inc., USA) and GRIN fiber (GIF625, Thorlabs Inc., USA). The spacer length provides the desired beam expansion inside the GRIN fiber and is needed due to the NA mismatch between the GRIN and SMF. The necessary NA and imaging range can be achieved by determining the appropriate spacer and GRIN fiber lengths.

The actual MFD value of the ER mode was estimated based on the following considerations. For video-rate navigation, a minimal frame rate $f_{\text{frame}}$ of 30 frames per second is required to ensure smooth and responsive operation. A tenfold average proved to be very beneficial to enhance image quality, and the spot size should be oversampled by at least a factor of four. Assuming a laser with a 3 MHz sweep rate $f_{\text{sweep}}$ is used, and the rectal circumference $C_{\text{rectum}}$ in the dilated state is ∼15  cm, the following spot size S is obtained:

$$S=\frac{C_{\text{rectum}}\cdot f_{\text{frame}}\cdot N_{\mathrm{os}}}{f_{\text{sweep}}}=\;\frac{15\;\mathrm{cm}\cdot 30\;\mathrm{Hz}\cdot 40}{3\cdot 10^{6}\;\mathrm{Hz}}=60\;\mu m,$$ (1)

where $N_{\mathrm{os}}$ denotes the oversampling factor. According to Eq. (2) and considering the focal lengths of the scan lens and collimator used in the rectoscope (as explained in Sec. 2.3), an MFD of ∼31  μm is required.

To determine the fiber length for achieving a 60  μm spot size after the imaging optics with the coreless and GRIN fiber combination, the light propagation through the fiber was simulated using a wide-angle beam propagation method implemented in MATLAB.^27^ The simulation showed that a coreless fiber between 150 and 300  μm and a GRIN fiber between 0.06 and 0.13 of the pitch length are needed. Given that the gradient constant of the GRIN fiber is $5.90\;{\mathrm{mm}}^{-1}$, this corresponds to a full pitch length of 1065  μm at a wavelength of 1300 nm. Therefore, a GRIN fiber length between 64 and 138  μm should be used.

A self-built fiber measuring device was used to measure the fibers and cut them to the required lengths. The final fiber assembly consisted of a 180  μm coreless fiber and a 94  μm GRIN fiber. To maintain the length of the GRIN fiber during polishing, an additional 600  μm of coreless fiber was added, but most of it was removed in the subsequent polishing steps. The 125  μm GRIN and SMF were then inserted into a 260  μm ferrule (30260C1, Thorlabs Inc., USA) as shown in Fig. 1(a), glued, and polished at an 8 deg angle to minimize lateral displacement between the fiber cores. The ferrule optic setup, along with the corresponding light beam profiles before and after passing through the imaging optics, is presented in Figs. 1(b) and 1(c).

### 2.2 Beam Characteristic Analysis of the Dual-Resolution Fiber Optic Ferrule

The MFD was quantified using a self-built system with LabVIEW software and a microscope consisting of a 10× objective (MSB50100, Nikon, Japan) and a camera (UI–1492LE, IDS Imaging GmbH, Germany). As expected, the measurements confirmed that the GRIN fiber probe had a wider MFD of 33.8  μm at its end compared with the standard SMF fiber, which had an MFD of 10.9  μm.

The spot size was also experimentally determined using the same setup but including the imaging optics employed in the rectoscope system described in Sec. 2.3. For this, the MFD was measured directly after the achromatic doublet scan lens, with the results shown in Fig. 1(d). In addition, the spot size $2\omega_{0}$, the Rayleigh length $z_{R}$, and the NA were calculated using the standard formulas for the Gaussian beam propagation:

$$2\omega_{0}=\mathrm{MFD}\cdot \frac{f_{S}}{f_{C}},\;z_{R}=\frac{\pi }{\lambda }\cdot \omega_{0}^{2},\;\mathrm{NA}=n_{0}\cdot \sin(\alpha ),$$ (2)

where $f_{S}$ is the focal length of the scan lens, $f_{C}$ is that of the collimator, and $n_{0}\approx 1$ is the refractive index of air. The half divergence angle α in the far field was determined using the measured MFD values. For the MFD value, the standard $1/e^{2}$ intensity definition of Gaussian optics was used instead of the 1% intensity definition sometimes used in datasheets for optical fibers, which can result in discrepancies. For example, the SMF-28 fiber is often specified with an NA of 0.14. However, based on the definition used in this paper, the NA is measured to be 0.09 at a wavelength of 1300 nm. The key spot characteristics obtained from these measurements are summarized in Table 1.

**Table 1:** Beam characteristics of the two fiber channels after the imaging optics.

Imaging mode	Measured spot size (μm)	Calculated spot size (μm)	Rayleigh length (μm)	Numerical aperture
HD mode (SMF-28 fiber)	19.0	21.0	265	0.09
ER mode (GRIN fiber probe)	62.8	65.3	2557	0.02

The 3D geometry of the two beam waists in relation to each other through the fully assembled rectoscope tip (described in Sec. 2.3) is shown in Fig. 1(e). The lateral separation between the two modes is ∼250  μm with an angle of ∼0.5  deg. The HD mode beam, in particular, exhibits astigmatism caused by the cylindrical lens effect of the exit window. These measurements were performed with a separate characterization setup using a 1060 nm light source to ensure sufficient sensitivity outside the focal plane.

### 2.3 OCT Imaging System and Rectoscope Design

The data presented in this study were obtained using a swept-source OCT system equipped with a commercial 3.2 MHz FDML laser (NG-FDML, Optores GmbH, Germany) operating at a center wavelength of 1310 nm. At a spectral bandwidth of 100 nm, the system has an axial resolution of ∼15  μm and an imaging depth range of ∼4.7  mm in air. Data acquisition was performed using a 12-bit analog-to-digital converter (ATS9373, AlazarTech, Canada) with a sampling rate of 4  GSa/s. The interference fringes were detected with a 1.6 GHz balanced photodetector (PDB480C-AC, Thorlabs Inc., USA).

**Fig. 2:**
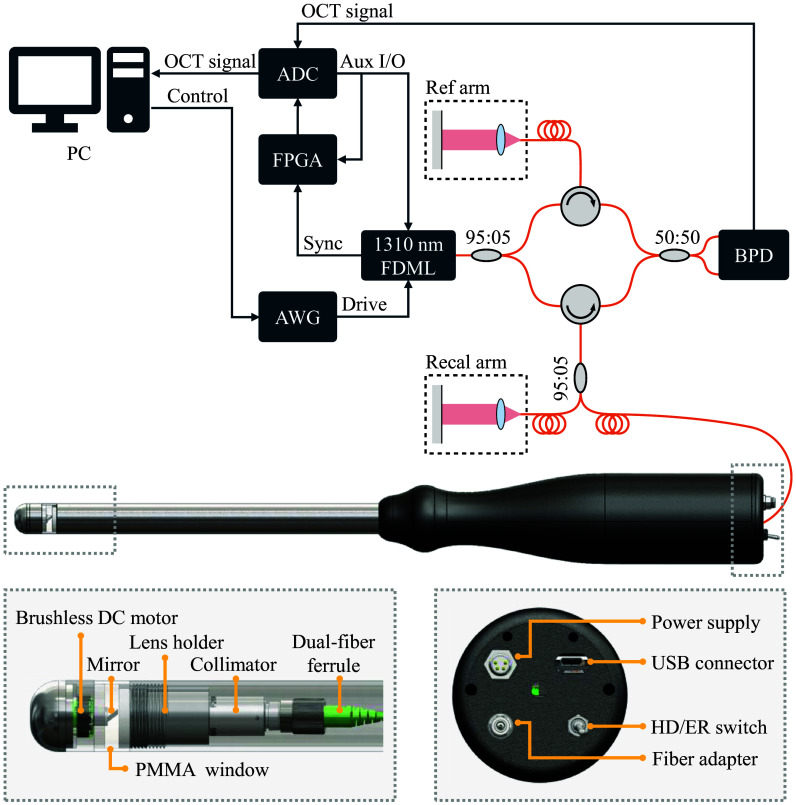
MHz-OCT experimental setup with rectoscope. The system diagram illustrates the structure of the interferometer, including the optical (orange) and signal (blue) paths. Important electronic components such as the arbitrary waveform generator (AWG), the field-programmable gate array (FPGA), and the analog-to-digital converter (ADC) are shown. The lower left image provides a zoomed view of the rectoscope tip, whereas the lower right image shows the back. PMMA, polymethyl methacrylate; HD, high-detail mode; ER, extended-range mode.

A self-developed rectoscope with an outer diameter of 20 mm was used in this study.^14^ A brushless ball bearing DC motor (1102, BETAFPV, China), a fiber collimator (F260APC-C, Thorlabs Inc., USA) with a focal length of 15.5 mm, and a 30 mm focusing lens (AC080-030-C, Thorlabs Inc., USA) are inside the distal end. The focused OCT beam is reflected by a 3 mm wide prism mirror (MRA03-M01, Thorlabs Inc., USA) mounted on the motor and emitted through a 1 mm thick polymethyl methacrylate (PMMA) tube, which serves as a transparent window for circumferential scanning; the prism is angled at 5 deg relative to the PMMA surface to minimize back reflections. A field-programmable gate array (FPGA, iCE40-HX8K, Lattice Semiconductor Corporation, USA) was used to synchronize the motor’s control system with the OCT system using a custom developed gateware. The motor controller in the probe uses the motor’s back electromotive force (BEMF) signal to determine its position using a virtual Hall sensing technique. It sends a rotational trigger signal to the FPGA to synchronize the motor’s position and initiate the image acquisition. This ensures precise timing and enables real-time imaging visualization.^14^ Although the mechanical housing and general probe architecture were retained from previous designs to ensure robustness and clinical compatibility, the optical configuration and functional capabilities were substantially extended in the present prototype. The focus was ∼375  μm outside the cylindrical optical window, with 22 mW of power applied to the sample. Figure 2 illustrates a schematic representation of the rectoscope and experimental setup.

In the 3D-printed handgrip, the electronic motor drive unit and a 1×2 MEMS fiber optical switch (MEMS-123221333, Agiltron Inc., USA) are encased. The fiber optical switch is controlled by a Teensy 4.1 microcontroller board (PJRC, USA), and imaging modes can be selected via a toggle switch on the handgrip. Based on the selected mode, the MEMS switch redirects the laser beam into the corresponding fiber channel. The switching time of 10 ms ensures minimal delay when transitioning between imaging modes.

Self-developed imaging software was utilized for real-time, low-latency imaging, facilitating instantaneous data visualization in Cartesian or polar representation. The imaging software determines the number of A-scans in real-time by using the BEMF signal. A screen recording of the software interface, demonstrating real-time operation and switching between the HD and the ER mode, is provided as Video 1 (MP4, 89.1 MB [URL: https://doi.org/10.1117/1.JBO.31.4.046002.s1]).

## 3 Results

### 3.1 Comparative Evaluation of the Imaging Range and Visual Clarity

To evaluate the extended imaging range capability of the rectoscope, four 3D-printed circular sawtooth targets of varying diameters were used. Fabricated from white polylactide (PLA) filament, they had inner diameters ranging from 25 mm (circumference ≈78.5  mm) to 60 mm (≈188.5  mm). A total of 8560 A-scans were acquired per revolution, with a motor speed set at ∼350  Hz. The laser bandwidth was set to 19.2 nm to increase the imaging range and facilitate a more accurate comparison of the two imaging modes. All images were processed using the same parameters. The color scale, shown in decibels (dB), is referenced to an arbitrary intensity level.

**Fig. 3:**
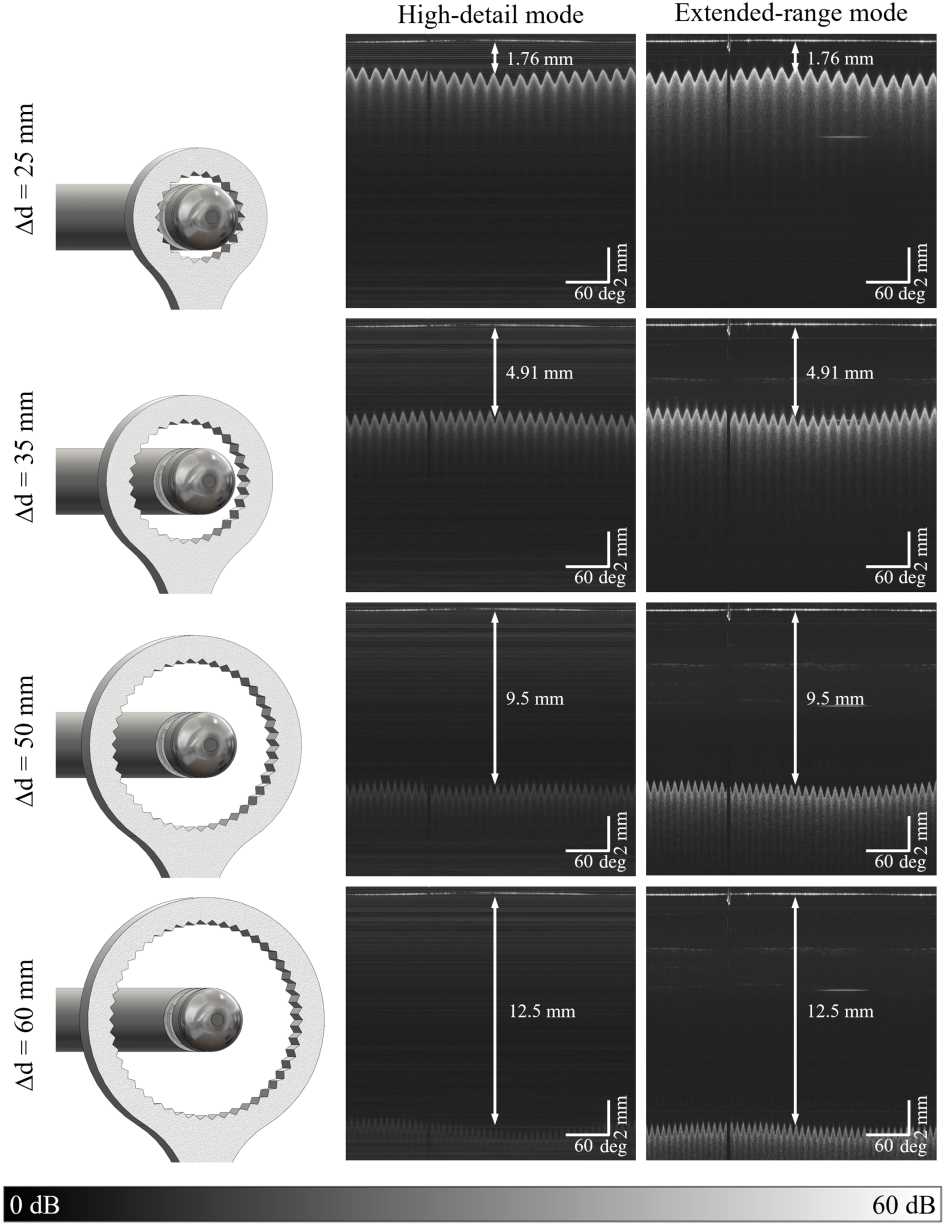
Experimental comparison of the extended-range and high-detail modes using 3D-printed circular targets. The front view of the rectoscope and the targets are illustrated on the left. Δd indicates the inner diameter of the circular sawtooth pattern in millimeters. The corresponding OCT B-scans (averaged intensity values over ten consecutive B-scans) of the high-detail (center) and extended-range mode (right) are shown. The laser bandwidth was set to 19.2 nm for both modes. The motor’s cable causes the black line observed in the OCT images. Identical processing parameters have been applied to all images. The color scale in decibels (dB) is relative to an arbitrary reference intensity.

The findings are presented in Fig. 3. On the left, the imaging setup is illustrated, showing the rectoscope positioned within the circular targets. In the center, the corresponding OCT B-scans, shown as averaged intensity values of ten consecutive B-scans, are presented for both the HD mode (middle) and the ER mode (right). The tips of the sawtooth pattern do not appear at the same height in the B-scans due to the nonconcentric placement of the targets relative to the endoscope. The cable of the brushless DC motor causes a black line to be visible in the OCT images.

The measurements show that even with the 25 mm target, the expected improvement in resolution provided by the HD mode was barely noticeable. This is likely because the targets were made of PLA filament, an unsuitable sample for distinguishing fine structural details. In addition, it can be assumed that the sample’s diameter was too large. With the focus set 374  μm beyond the endoscope window and the target positioned 1.76 mm outside, it was markedly out of focus. Furthermore, the results indicate that the HD mode could detect targets with a tip distance of up to 35 mm, which equals 4.91 mm outside the endoscope window, although the signal intensity was low. By contrast, the ER mode exhibited noticeably higher signal intensity with the same target.

For the 50 and 60 mm targets, structures in the HD mode became increasingly difficult to distinguish, demonstrating its limitation in visualizing deeper tissue structures. However, with the ER mode, the sawtooth pattern remained distinguishable even at 12.5 mm from the endoscope window. This capability underscores its potential value for clinical applications where comprehensive visualization of the rectal wall layers is required to evaluate the entire rectal circumference.

The results demonstrate that the ER mode markedly enhances the differentiation of various structures, especially when the target sample is not in direct contact with the rectoscope. This is due to the increased Rayleigh length, which ensures that the beam maintains its focus over a greater distance, allowing for more detailed imaging of structures further away from the rectoscope. This capability makes it valuable for the preliminary identification and localization of significant abnormalities during medical examinations.

### 3.2 Dual-Resolution OCT Imaging of the Postmortem Human Rectum *In Situ*

Imaging was performed in the rectum of two female body donors (body donor 1: 92 years, 158 cm, 43 kg; body donor 2: 93 years, 156 cm, 69 kg) who were recruited from the local body donation program (Institute of Anatomy, Kiel University, Germany) after previous informed written consent to be used for medical research and educational purposes and approval by the local ethics committee (D514/24, Medical Faculty, Kiel University). All studies with postmortem human biomaterial strictly followed guidelines and rules to maintain ethical (No. D 465/24) and scientific standards. To preserve normal tissue consistency, the bodies were fixed using an ethanol-glycerol-lysoformin solution injected through the femoral arteries (postmortem interval to fixation: body donor 1, 35.5 h; body donor 2, 94 h).^28^ This solution consisted of 69.7% ethanol, 30% glycerol, and 0.3% lysoformin, with a volume of ∼0.3  L/kg of body weight. Standard macroscopic sigmoidoscopy using a colonoscope (CF-HQ190L/I, Olympus, Japan) connected to a compatible endoscope processor (EVIS-Exera-III, CV-190-Plus, Olympus, Hamburg, Germany) confirmed that the ethanol-glycerol-lysoformin fixation method effectively preserved the rectum’s native anatomy and tissue consistency, providing excellent conditions for examination by maintaining both flexibility and mucosal integrity. After fixation, the colon was internally rinsed with water, and the rectosigmoid junction was tied off via a transabdominal approach to ensure the cleanliness of the mucosal surface. The rectum was examined endoscopically in lithotomy position.

To facilitate comparison, OCT B-scans were obtained with both resolution modes at nearly identical positions. Each B-scan comprised ∼8860 A-scans, captured with a motor speed of ∼350  Hz and a laser bandwidth of 95 nm. It is essential to note that, despite applying averaging, imaging was conducted at low-latency video rates (∼25 averaged frames per second), ensuring high image quality and low jitter performance, as demonstrated in Video 1. However, given that the data collected with the rectoscope probe exhibited a small angular position deviation of ∼2  mrad, the B-scans were registered using a 2D cross-correlation method after imaging and during postprocessing.^29^ Following registration, the images were averaged using the intensity values of ten consecutive B-scans.

#### 3.2.1 Impact of lateral resolution on rectal tissue visualization

**Fig. 4:**
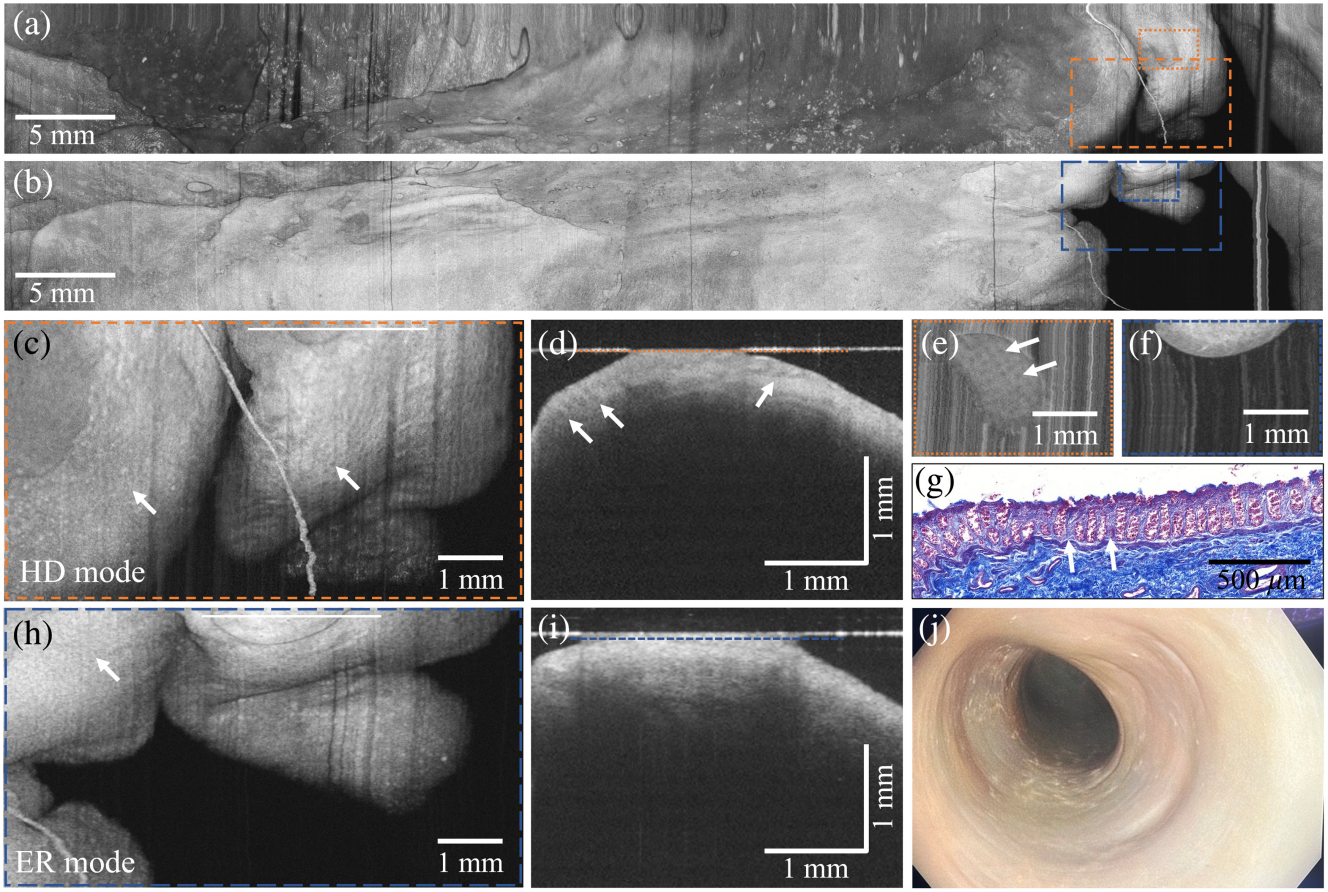
Manual pullback OCT imaging of a human postmortem rectum *in situ*. Standard deviation *en face* projections of all layers acquired in the high-detail (HD) mode (a) and the extended-range (ER) mode (b) show consistent rectal surface regions. Zoomed-in *en face* views of two corresponding regions, marked by orange and blue dashed outlines in panels (c) and (h), reveal a typical dotted crypt pattern (white arrows), which is more distinctly resolved in the HD mode. In both *en face* projections, white lines indicate the positions of the corresponding B-scans (averaged intensity over ten consecutive frames) shown in panels (d) and (i). Superficial *en face* cross-sections (averaged intensity over ten consecutive depth layers) are displayed in panels (e) and (f); these were extracted just beneath the glass surface, as indicated by dotted lines in panels (d) and (i). In the cross-sectional *en face* views and B-scans, crypt structures are visible only in the HD mode; see panels (d) and (e). (g) Azan-stained histological section of the rectal wall from a separate sample for comparison with the OCT findings. (j) Endoscopic image acquired from approximately the same location for anatomical reference.

To evaluate the benefit of increased lateral resolution, manual pullbacks were performed in the rectum of body donor 2 using both imaging modes of the rectoscope. The resulting datasets are summarized in Fig. 4. Large-area *en face* projections from the HD [Fig. 4(a)] and ER [Fig. 4(b)] modes demonstrate comparable overall coverage of the rectal surface, allowing direct visual comparison of image characteristics. Representative regions are shown at higher magnification [Figs. 4(c) and 4(h)] together with corresponding cross-sectional B-scan views [Figs. 4(d) and 4(i)] and superficial *en face* slices [Figs. 4(e) and 4(f)] extracted from the same volumes. An Azan-stained histological section from a separate sample [Fig. 4(g)] is provided for structural correlation, and a conventional endoscopic image from approximately the same location serves as anatomical reference [Fig. 4(j)].

It should be noted that the pullbacks were performed manually. Although corresponding regions were selected for comparison between the two imaging modes, variations in pulling speed and hand motion led to slight differences in probe trajectory and sampling. Therefore, minor changes in the apparent shape, size, or position of tissue features do not necessarily represent true anatomical differences, and the exact spatial extent of the pullback cannot be determined retrospectively. In addition, the rectal wall is compliant and can deform between pullbacks, so folds and surface patterns may appear somewhat different. Nevertheless, the large-area projections confirm that the same general tissue regions were visualized in both modes.

Compared with standard endoscopy, the OCT *en face* projections provide markedly higher structural contrast of the mucosal surface and subsurface architecture. Fine surface patterns are visible that are not discernible in the endoscopic image. In selected regions, a dotted texture consistent with mucosal crypt architecture (indicated by arrows) can be appreciated in the OCT *en face* views [Figs. 4(c) and 4(h)] and is supported by the corresponding histological section [Fig. 4(g)].

Despite the clear difference in imaging range (Fig. 3), the difference in lateral resolution between the HD and ER modes in the OCT *en face* projections is less pronounced than might be expected from the nominal MFD [Figs. 4(c) and 4(h)]. This can be attributed to several factors. First, the Rayleigh length primarily affects the depth over which the beam remains focused, whereas the lateral resolution at the focal plane differs less dramatically. Second, tissue scattering, refractive index variations, and optical aberrations introduced by the cylindrical window reduce the practical difference between the nominal MFD. As a result, both modes provide a comparable overall depiction of tissue morphology, with the HD mode mainly enhancing image sharpness and contrast of fine features rather than revealing entirely new structures.

Nevertheless, the HD mode consistently reveals slightly sharper boundaries and finer textural detail, particularly in superficial layers [Fig. 4(e)]. For example, crypts can be faintly observed in the ER mode *en face* projections [Fig. 4(h)], but they are more clearly delineated in the HD mode [Fig. 4(c)]. In the HD datasets, the cross-sectional crypt outline can also be identified in the corresponding B-scans [Fig. 4(d)], whereas they are not visible in the ER mode [Fig. 4(i)].

#### 3.2.2 Tissue characterization and histological validation

**Fig. 5:**
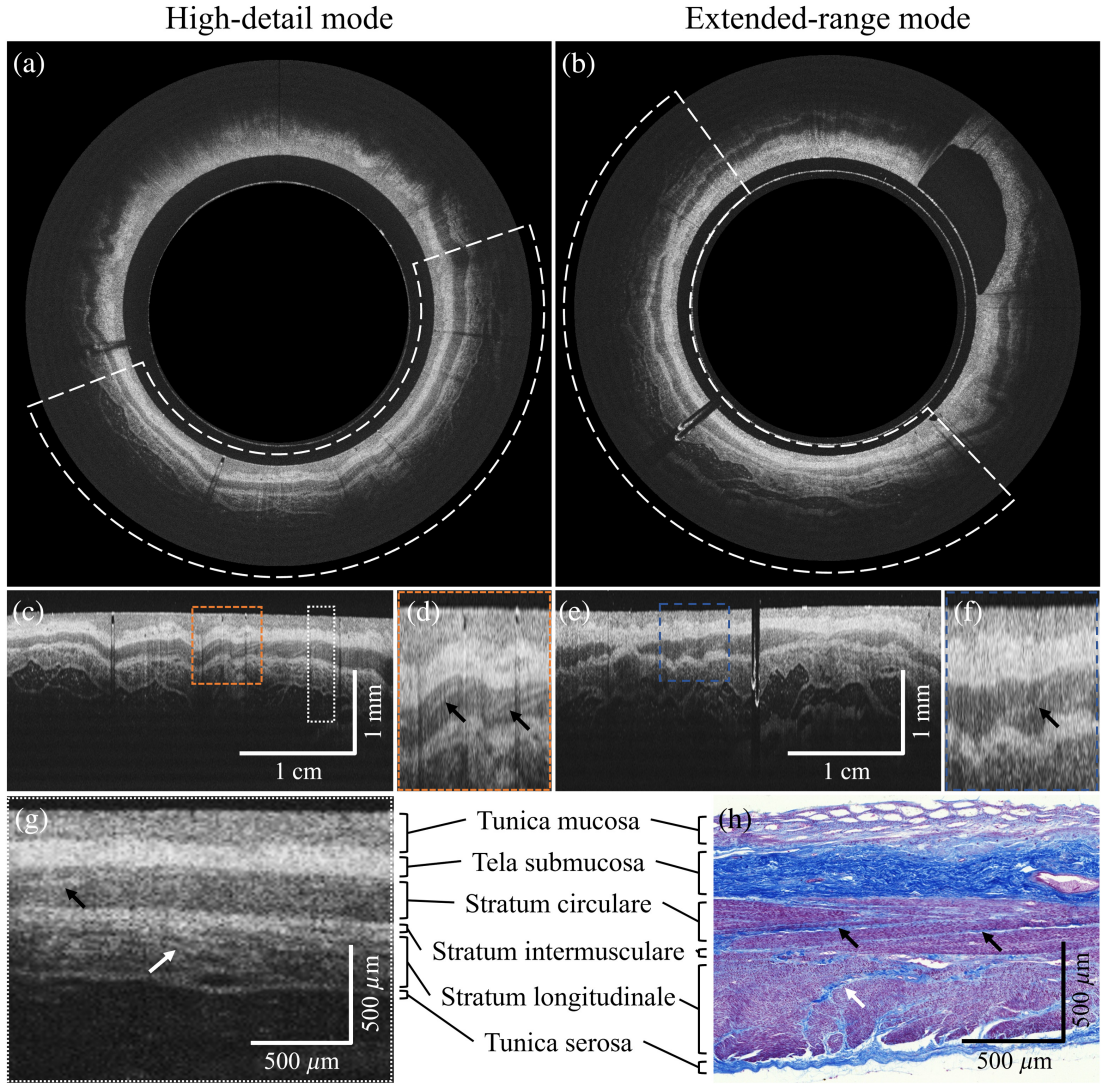
Endoscopic OCT of a human postmortem rectum in situ. B-scans were registered and averaged using the intensity values of ten consecutive B-scans. Polar views of (a) the high-detail (HD) and (b) the extended range (ER) mode. (c) and (e) Enlarged Cartesian views of white-dotted areas in the polar view. In panels (d) and (f), zoomed-in views of the orange-dotted rectangles in panels (c) and (e) are shown. Bright stripe-like features in the muscular layers correspond to intramuscular connective tissue septa, which are less distinctly resolved in the ER mode. (g) Magnified Cartesian view of the HD mode [white-dotted area in panel (c)], allowing detailed characterization of the rectal wall layers. (h) Azan-stained histologic section from approximately the same position. Black arrows indicate septa in the stratum circulare, and white arrows indicate septa in the stratum longitudinale, corresponding to the stripe-like features observed in the OCT images.

To verify the rectal tissue structures visualized with OCT, imaging was followed by subsequent tissue excision and histological examination of the corresponding tissue sample. The OCT data and histological sections presented here were both obtained from body donor 1. Figure 5(a) shows the HD mode in the polar view, with zoomed-in Cartesian views presented in Figs. 5(c) and 5(d). Similarly, Fig. 5(b) displays the ER mode in the polar view and zoomed-in Cartesian views in Figs. 5(e) and 5(f). Figure 5(g) provides a zoomed-in view of the white-dotted area in Fig. 5(c). After imaging, a small rectal tissue sample was excised and stained with Azan. The corresponding section is shown in Fig. 5(h).

With OCT images from both modes, the four primary layers of the rectum (mucosa, submucosa, muscularis propria, and serosa) could be clearly discriminated. The mucosa appeared as a broad, pale gray band. The submucosa was visualized as an inhomogeneous, light gray broad area displaying several dark spots corresponding to the submucosal blood vessels. Furthermore, the muscularis propria, which comprises the circular and longitudinal muscle layers, was characterized by two homogeneous dark gray bands separated by a light gray intermuscular plane. The serosa was displayed as a bright band. However, the ER mode could clearly identify tissue structures beyond the tunica serosa that are either not or only marginally discernible with the HD mode. This improved visibility is due to the increased detectable depth signal resulting from the longer Rayleigh length.

In the magnified views in Figs. 5(d) and 5(f), which highlight the areas marked in orange and blue in the corresponding Cartesian views, bright stripes are visible in the stratum circulare in the HD mode. These stripes, highlighted by black arrows, correspond most likely to connective tissue septae, which are typically found within the muscularis propria to serve as an anchoring scaffold for smooth muscle cells, as evidenced in the histology section [Fig. 5(h)]. As the axial resolution remains unchanged between modes, these features could also be identified in the ER mode, although with less clarity due to the lower lateral resolution.

In general, the tissue images were more detailed regarding the granularity and the resolution of the mucosa than those of an average EUS system used in clinical routine.^30^ This evaluation was performed by two experienced endoscopists (B. S. and M. E.) using a subjective five-point visual analogue scale (5 OCT more detailed than EUS, 4 OCT slightly more detailed than EUS, 3 equal, 2 OCT slightly less detailed than EUS, 1 OCT far less detailed than EUS). Although EUS provides an adequate spatial overview of the intestinal wall and captures larger structures at greater depths, it lacks the resolution necessary to visualize fine tissue features. By contrast, the MHz-OCT system offers high-resolution imaging with clear separation of individual wall layers, particularly the mucosal sublayers such as the epithelium, the lamina propria, and the lamina muscularis mucosae, which are not distinguishable using conventional EUS.

#### 3.2.3 HD mode: detection of the lamina muscularis mucosae

**Fig. 6:**
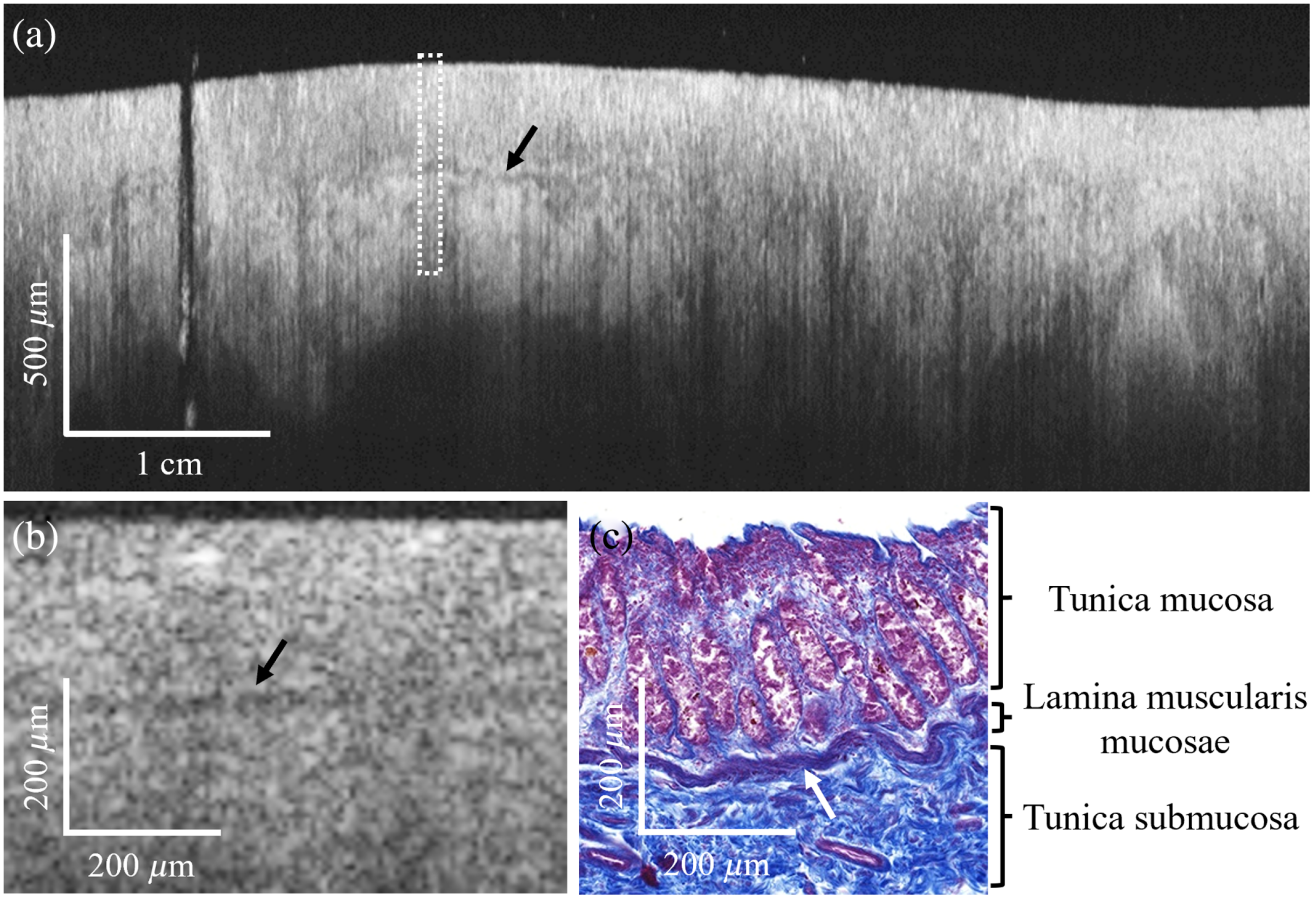
OCT imaging of the lamina muscularis mucosae (LMM) in a postmortem human colon with the high-detail mode. (a) OCT rectoscope image showing the LMM (averaged intensity over ten consecutive frames). (b) Enlarged view of the white outlined rectangle. (c) Azan-stained histological section of the LMM, derived from a separate sample, provided for comparison with the OCT imaging results. Arrows indicate the position of the LMM.

The HD mode proved especially useful for detecting the lamina muscularis mucosae (LMM), as shown in Fig. 6(a). This thin muscle layer separates the tunica mucosa from the tela submucosa. It is discernible as a distinct, slightly meandering band of reduced signal intensity readily distinguishable from the adjacent mucosal tissue displaying a brighter and increased signal intensity. A magnified view in Fig. 6(b) highlights this layer more clearly, with a black arrow indicating its position.

Figure 6(c) shows a corresponding Azan-stained histological section obtained from a separate, serially processed tissue sample to confirm the OCT-based identification of the mucosal layers. The LMM is discernible in histology as a thin, red-stained meandering band extending between the mucosal epithelial-rich crypts and blue-stained connective tissue of the submucosa. This obvious correlation supports the validity of the OCT findings and highlights the system’s ability to delineate fine anatomical structures within the intestinal wall. As a note, while crypts are visible in the corresponding histological section, they cannot be distinguished in this OCT B-scan. This likely reflects the limited lateral resolution of the system, the intrinsic difficulty of resolving crypts in OCT B-scans,^7^ and the fact that the OCT and histology sampling were performed at different tissue locations with potentially lower crypt density.

The LMM could not be visualized in the ER mode. As a result, no data from this layer could be acquired using that imaging mode, highlighting the strength of the dual-fiber ferrule design. The high-resolution imaging becomes especially important in the context of tumor staging. When tumor cells extend beyond the LMM and reach the submucosa, the risk of further progression increases. The submucosa contains numerous blood and lymphatic vessels, making it a critical pathway for the spread of cancer cells. This invasion is associated with a greater likelihood of lymph node metastasis and distant tumor formation.^31^ The ability to identify the LMM may therefore serve as an important diagnostic marker for detecting early invasion. Although the perceived difference in image quality between the two imaging resolutions is not that drastic, the unequivocal identification of the LMM emphasizes the benefit of resolution switching allowing for high-resolution diagnostic capabilities.

## 4 Summary and Outlook

This work presents the design and application of a dual-resolution mode rectoscope prototype using a 3.2 MHz-OCT system for *in situ*, live, and low-latency imaging of a human rectum. The OCT rectoscope integrates a GRIN fiber probe for an extended imaging range and a SMF for higher-detail imaging within a single distal ferrule. The dual-mode concept allows real-time switching between modes depending on imaging requirements.

Compared with state-of-the-art EUS^30^ and our earlier rectoscope prototypes,^9^^,^^14^ the current system achieves markedly improved image quality. Key technical changes include a reduction in motor speed from 667 to 350 Hz and the use of a collimator with increased focal length, which reduced A-scan spacing from ∼13.4 to 7  μm and decreased the spot size in air after the imaging optics from ∼35 to 21  μm. These improvements allow clearer resolution of inner layers, as confirmed by experienced anatomists (T. H., M. H., T. W.), who identified all major rectal wall layers in the OCT images, including the tunica mucosa, tela submucosa, circular and longitudinal muscle layers, and tunica serosa. The lamina muscularis mucosae, a thin muscle layer separating the mucosa from submucosa, as well as crypt-like features, could only be clearly visualized using the HD mode, highlighting the added value of higher-resolution imaging for fine anatomical structures. By contrast, the ER mode provides a substantially longer effective imaging range due to its extended Rayleigh length, allowing visualization of the full rectal circumference and maintaining higher signal intensity in deeper tissue layers, which is advantageous for rapid overview imaging and comprehensive assessment of the rectal wall. Together, these complementary capabilities demonstrate the practical advantage of on-the-fly resolution switching.

Interestingly, despite the pronounced differences in nominal MFD and Rayleigh lengths, the perceived difference in lateral resolution between HD and ER modes is surprisingly modest. Most tissue structures, including mucosal folds and major wall layers, are already clearly visible in ER mode, demonstrating that even the lower-resolution mode provides fairly high-quality structural images. The relatively small observed difference is partly due to astigmatism introduced by the exit window, which reduces the effective resolution, especially of the HD mode. In future designs, this could be mitigated, for example, by using a thinner PMMA tube and optimizing the optical design, which would enhance the distinction between the two modes.

Postmortem changes, including alterations in tissue refractive index due to early autolysis, may potentially affect OCT image quality. In this study, the specimens were fixed 35.5 h (body donor 1) and 96 h (body donor 2) postmortem. Despite these differences in postmortem interval and the presence of early autolytic changes, the OCT image quality was comparable between the two donors. In addition, the exceptionally high image quality is supported by the use of ethanol-glycerol-lysoformin-fixed donor tissue, which preserves both anatomy and tissue contrast effectively and may contribute an “optical clearing” effect, further enhancing OCT signal quality. Whether similar image quality can be achieved in nonfixed tissue *in vivo* remains to be systematically evaluated in future studies.

Given the challenge of imaging the complete, unfolded rectal circumference and preventing the tissue wall from shifting out of the optimal focal plane during *in vivo* live navigation, future developments will incorporate a balloon-mounted rectoscope to provide stable imaging conditions. This balloon would expand the rectum, ensuring broad contact between the rectal wall and the rectoscope, particularly when using the ER mode. In addition, an expanded rectum leads to an increased circumference that must be scanned completely. In this case, using a small spot size would result in undersampling or prolonged acquisition times, as the scan density across the larger circumference decreases. Therefore, a larger spot size is required to maintain an adequate scanning density while preserving the scanning speed. Furthermore, a balloon could facilitate pullbacks to acquire 3D OCT datasets of the rectum. This pullback procedure could be performed manually or controlled using a motorized pulling mechanism.^32^

Although high imaging speed is a key enabler for practical large-area and real-time endoscopic OCT, broader clinical adoption will ultimately depend on the validation of robust, disease-specific OCT-derived biomarkers and their integration into established diagnostic workflows. The rectal tissue of the body donors showed no macroscopic pathological findings, and thus the presented data represent normal rectal wall morphology. Nevertheless, the ability to inspect volumetric OCT data at multiple depths highlights the potential of this approach for clinical tasks such as tumor staging, where accurate delineation of layer boundaries and invasion depth is critical. In such scenarios, even the modest gain in lateral resolution provided by the HD mode may improve diagnostic confidence, whereas the ER mode remains advantageous for rapid overview imaging of larger areas. Beyond rectal imaging, the presented approach could be extended to the diagnosis and monitoring of other gastrointestinal pathologies, e.g., inflammatory bowel diseases, including the assessment of the intra- or transmural extent of inflammatory processes. Although the current system is implemented as a rigid rectoscope, the active imaging components are confined to the distal probe tip, whereas the optical and electrical connections are inherently flexible, allowing future realization in a colonoscope-like form factor or as a tethered capsule that can be pulled through the entire colon.

Overall, the dual-resolution MHz-OCT rectoscope presented in this study demonstrates strong potential as a versatile, noninvasive diagnostic tool for on-site assessment of gastrointestinal disease and therapeutic response.

## Supplementary Material

10.1117/1.JBO.31.4.046002.s1

## Data Availability

The original data used to generate the results reported in this paper are not publicly available due to their size. They can be obtained from the authors upon reasonable request.
